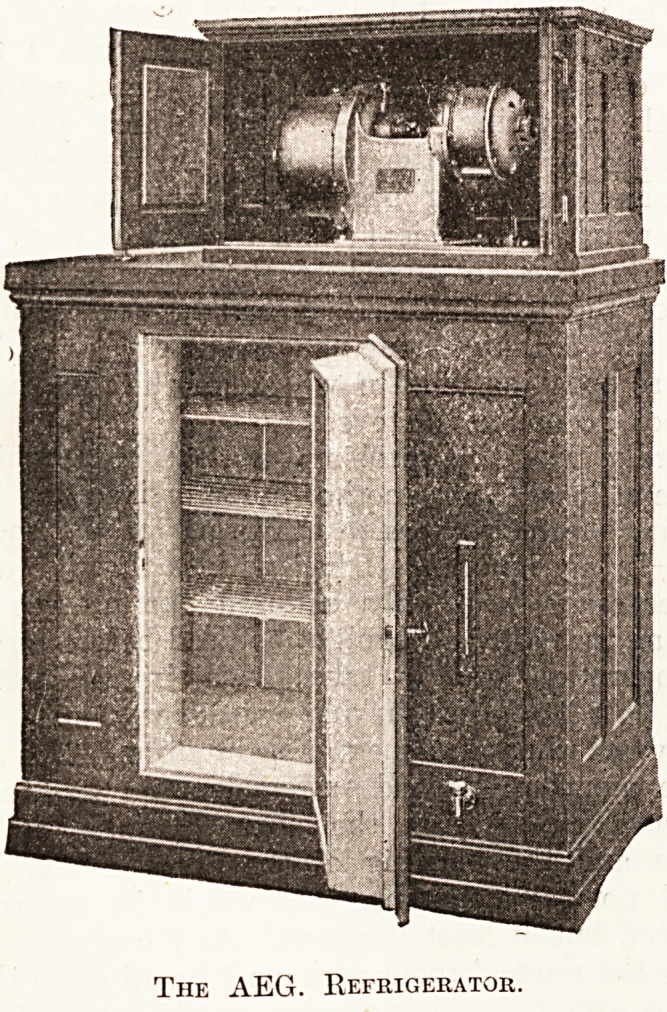# Institutional Needs

**Published:** 1914-06-20

**Authors:** 


### INSTITUTIONAL NEEDS.

### AN IMPROVED MOUTH-GAG.

A mouth-gag which is specially adapted for use in ear and throat work and for dental surgeons has been designed by Frederick W. Sydenham, M.D., F.R.C.S.

Edin., of Walsall, Staffs. This gag has certain advantages over the existing ones, and will no doubt supply a genuine want. The chief features are: (1) the limbs of the cheek portion are long, the joint being set well back, an essential in easy enucleation of the tonsils by Sluder's method; (2) the ratchet method of opening gives great force, with a gradual yet rapid opening of the mouth, a desideratum when operating without an anæsthetic: there is also less danger of breaking or extracting the teeth; (3) there are no projections, as the thumb-piece which operates the ratchet is provided with a tumble-joint, permitting it to lie flat against the cheek; and (4) the alveolar projection is at an acute angle, so that it enters the mouth between the incisor teeth at right angles to them, giving the gag a firm hold, with less tendency to slip than is usually the case. The design has been carried out by Messrs. Allen and Hanburys, Ltd., of Wigmore Street, London, W., from whom it may be procured. The accompanying illustrations demonstrate the peculiar characteristics of Mr. Sydenham's gag.

### TONIC FOODS AND HOSPITAL STAFFS.

Ever since the modern proprietary medicine made its appearance, pure barley malt has been recognised as a basis for tonic foods, in the same way as cod-liver oil has been used as a basis for nutrients. Among the former, as its name implies, "Biomalz" is noteworthy as a simple and easily assimilated food, which is of value in arresting the breakdown that so often follows upon overwork in institutions, and is, in fact, of frequent use in them among the staff as well as the patients. The health problems involved in the changes from day to night duty and back again, to say nothing of those recurrent periods of depression to which everyone is liable in a changeable climate like ours, necessitate not merely very carefully prepared meals and a balanced diet, but something in addition to fall back upon which is not a stimulant. This something may very well be "Biomalz." Requiring no preparation, and equally convenient in the vehicle of milk, tea, cocoa, coffee, or soup, children and adults may alike benefit from it. The manufacturers are Peterman Bros., Chemical Works, Regent House, Kingsway, London, W.C., and now that it is getting a hold among the staffs of institutions, its further extended use is hardly a matter of doubt. Maltose, water, dextrine, nitrogenous matter, and phosphates, are its main constituents, and, being very palatable, there is no difficulty in inducing patients to take it readily.

### MODERN METHODS OF REFRIGERATION.

Cold, or, in scientific language, absence of heat, can be produced either by melting ice or by mechanical refrigeration. Mechanical refrigeration, of course, is based on the fact that a liquid which evaporates absorbs heat from its surroundings. The liquids used in existing commercial refrigerators are chemical substances easily evaporated, such as carbon dioxide, ammonia, and sulphur dioxide. In the AEG refrigerator æthyl chloride is used. A refrigerator is a device consisting of various parts so connected that the vapour produced by the evaporation of the liquid is recovered by compressing and condensing it, the substance being used over again. The heat absorbed during evaporation is given out during compression and condensation, and must be taken away by circulating cooling water. An air space is cooled by placing the part in which evaporation takes place into it. Ice is produced indirectly by cooling a brine bath in which the water-vessel is im-

The AEG. Refrigerator.

mersed. To come to the description of this type of cooling chamber, it may be said that the AEG refrigerator consists of an ice-chest in which the machine proper is placed; the latter comprises a copper coil (the upper portion of which forms a closed vessel) in which the evaporation takes place. The lower part cools the chest, while the upper part contains the brine-bath used for ice-making. There is also a compressor "aspirating" the vapour on its suction side and compressing it. The "condenser" is placed in the base of the machine. Finally, there is a "reducing valve," through which the liquid enters the refrigerator, the pressure suddenly dropping, and a "motor" totally enclosed for alternating or direct current. The only operations necessary in starting the machine are turning on the cooling-water supply and starting the motor, the machine being automatic. Power consumed is given as only $\frac{1}{4}$ h.-p., and the cooling-water consumed is only about 9 gallons per hour. The AEG refrigerator has a capacity of 880-1,000 B.Th.U. per hour, which value will be appreciated if it is remembered that a pound of ice in melting absorbs 144 B.Th.U. The AEG refrigerator is designed to perform simultaneously the following work: Cooling the air contained in the chest (127 cubic feet) to about 24° F., and making about 2.2 lb. of ice per hour. The actual performance is dependent on the air temperature, whether the door is opened frequently or not, whether the brine is clean, or whether the cooling-water supply is ample. It is obvious that the advantages of mechanical refrigeration are mainly that it can be used where ice cannot be obtained or where its price is excessive; that lower temperature can be obtained; that pure artificial ice can be made for cooling drinks; while the cooled air is dry, as all moisture is frozen out, and the risk of putrefaction or mildew is minimised. It is always ready for immediate use.

### BARLEY WATER FOR HOSPITAL STAFFS.

In Mr. Shaw's "Cæsar and Cleopatra" Cæsar is represented as drinking barley water in preference even to Falernian wine. As that of a man of genius this preference is noteworthy. It is shared by many lesser men. When made with Robinson's "Patent" barley it is a capital drink for the athlete and the institutional worker, both for him who is always exercising his limbs in the open air, and for him who spends hours in hospital, for it is stimulating as well as refreshing. Mr. Hammond, a former chef at the Bachelors' Club, is credited with the following recipe: "Put the outside peel of two lemons into two quarts of water, add eight lumps of sugar, and boil for ten minutes. To this add two dessert spoonfuls of Robinson's "Patent" barley, previously mixed to a smooth paste with a little cold water. Continue to boil for five minutes and allow to cool. When cold strain off through fine muslin and add ice and lemon-juice to taste." It also forms in suitable cases an excellent adjunct to the sick room, and one which when well made does not pall on the patient.

### A RECORD HOSPITAL EGG-WEEK.

We are informed that the total number of eggs collected this year in connection with Hospital Egg-week amounts to 38,000, which constitutes a record. Previously it had not proved possible to reach more than 27,000 eggs, and therefore there is now a prospect that with 50,000 as the next round number to aim at a substantial aid to hospital housekeeping will be forthcoming. Thanks are due to Mr. F. Carl for his share in the undertaking, and if he could only impress upon the public mind the truth for this purpose underlying Samuel Butler's profound dictum that "A hen is only an egg's way of making another egg," even 100,000 might be raised in a year or two.

## Figures and Tables

**Figure f1:**
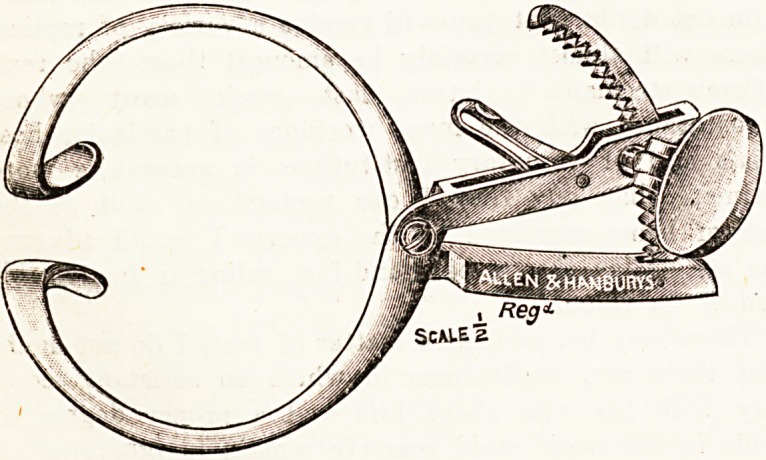


**Figure f2:**
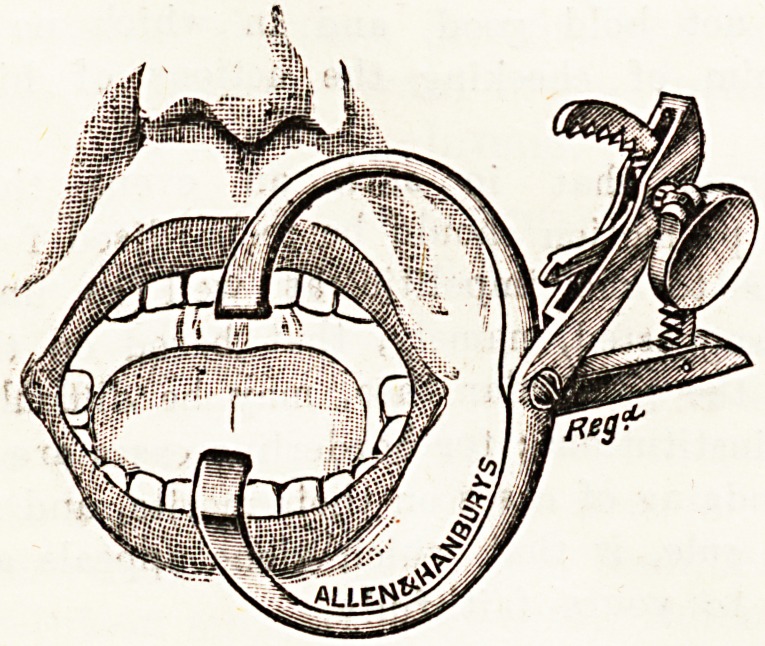


**Figure f3:**